# Molecular and Biological Properties of Snakins: The Foremost Cysteine-Rich Plant Host Defense Peptides

**DOI:** 10.3390/jof6040220

**Published:** 2020-10-12

**Authors:** Tao Su, Mei Han, Dan Cao, Mingyue Xu

**Affiliations:** 1 Co-Innovation Center for Sustainable Forestry in Southern China, College of Biology and the Environment, Nanjing Forestry University, Nanjing 210037, China; sutao@njfu.edu.cn (T.S.); qiuqiu121156291@outlook.com (D.C.); mingyuexu0116@163.com (M.X.); 2 Key Laboratory of State Forestry Administration on Subtropical Forest Biodiversity Conservation, Nanjing Forestry University, Nanjing 210037, China

**Keywords:** snakin, gibberellic acid stimulated in arabidopsis (GASA), plant host defense peptides (HDPs), antimicrobial peptides (AMPs), hormone

## Abstract

Plant host defense peptides (HDPs), also known as antimicrobial peptides (AMPs), are regarded as one of the most prevalent barriers elaborated by plants to combat various infective agents. Among the multiple classes of HDPs, the Snakin class attracts special concern, as they carry 12 cysteine residues, being the foremost cysteine-rich peptides of the plant HDPs. Also, their cysteines are present at very highly conserved positions and arranged in an extremely similar way among different members. Like other plant HDPs, Snakins have been shown to exhibit strong antifungal and antibacterial activity against a wide range of plant pathogens. Moreover, they display diversified biological activities in many aspects of plant growth and the development process. This review is devoted to present the general characters of the Snakin class of plant HDPs, as well as the individual features of different Snakin family members. Specifically, the sequence properties, spatial structures, distributions, expression patterns and biological activities of Snakins are described. In addition, further detailed classification of the Snakin family members, along with their possible mode of action and potential applications in the field of agronomy and pathology are discussed.

## 1. Introduction

Plants as sessile organisms are constantly facing threats from a wide spectrum of microorganisms, including bacteria, fungi, viruses and protozoa, as well as herbivores and insects; they have evolved highly effective mechanisms to defend against invaders that are harmful to their life. Plant host defense peptides (HDPs), also known as antimicrobial peptides (AMPs) are regarded as one of the most prevalent barriers elaborated by plants to combat these microorganisms in a rapid, direct and durable way [1]. Plant HDPs are ancient weapons of defense, constituting essential components of plant innate immune systems [1,2,3]. HDPs are arbitrarily referred to as small, thermal stable and positively charged peptides, generally comprising peptides of less than 100 amino acid residues with an overall net charge of +2 to +9, and molecular weight of 4 to 9 kDa [4,5]. They also possess a considerable proportion of hydrophobic amino acids (>30%) within a linear or cyclic structure [6]. HDPs have a broad spectrum of antifungal, antibacterial, antiviral and anticancer activities (see reviews by [4,7,8,9,10,11]). While most HDPs function in host defense as direct microbicides, others act as modulators that indirectly regulate the host immune response [12]. HDPs can restrain or kill pathogenic organisms at micro-molar concentrations, commonly by non-specific mechanisms [13]. This potentially helps HDPs to evade resistance development in target organisms, which makes HDPs a promising alternative to conventional antibiotics [9].

In past decades, many studies that attempted to explore the mode of action of HDPs were described. It has been demonstrated that the general activity mechanism of HDPs is associated with disruption of the cell membrane. Many HDPs are polycationic, whereas the lipids on the surface of bacteria are mainly anionic; thus, disruption or clustering of anionic lipids occurs when cationic peptide binds to the surface of the microbial membrane. In fungal or yeasts cell walls/membranes, the interaction with HDPs involves negatively charged components such as glycophospholipids (GPLs), sphingolipids and mannoproteins [14]. The interaction consequently can cause membrane disruption, membrane permeation, pore formation, ion channel modification, dissipation of electrochemical gradients, leakage, and/or eventual cell death. Many reviews were published on this subject [6,10,13,14,15,16,17,18,19]. Although membrane interactions are a main contributor to the activity of most HDPs, there are accumulating reports of HDPs that act on intracellular targets [8]. So far, at least four types of action mechanism of HDPs have been reported [11]: (1) HDPs directly interact with microbial membranes, resulting in membrane fluctuation, pores formation and/or membrane depolarization, finally dysfunction of membranes and cell death; (2) HDPs impede the biosynthesis of intra- and/or extra-cellular biomolecules, such as proteins, nucleic acid lipoteichoci acids and galcopeptides, causing metabolism abnormalities; (3) HDPs increase the K^+^ efflux and disrupt Ca^2+^ homeostasis and reactive oxygen species (ROS) production, leading to cell apoptosis; (4) HDPs inhibit adenosine triphosphate (ATP) synthesis, resulting in blocking ATP-dependent cellular processes.

The antifungal mechanisms of antifungal peptides/proteins (AFPs) have been well characterized [14]. AFPs can either interact with cell receptors inducing signaling cascades accompanied by Ca^2+^ influx, and/or translocation to fungal membrane via transporters [20]. The antifungal activity of AFPs is generally peptide- and species-dependent. For instance, although the radish defensins strongly inhibit the growth of certain fungi, it has no influence on other species [21]. The antifungal potency is determined by several parameters, such as the (cationicity) net charge, the hydrophobicity, the distribution of the residues and associated structure, length, and amphipathicity [22,23]. Moreover, HDPs can penetrate the cells and interact with inner cell compartments like mitochondria and stimulate cell apoptosis [24].

In plants, the majority of HDPs are cysteine-rich, and the number of cysteine residues is even, for example 2, 4, 6, 8, 10 and 12 [25]. This feature elicits the formation of multiple disulfide bonds, conferring plant HDPs with extraordinary high thermal, chemical and proteolytic stability which is crucial for them to act as a chemical shield against multiple pests and pathogens [26,27]. The distinctive topological configuration facilitates HDPs to sustain their conformation and activity even in harsh environments, such as low pH (e.g., plant vacuole) or proteolytic (e.g., the digestive systems of herbivores) conditions (for reviews see [27]). HDPs are scattered widely in nature, including mammals, birds, insects and plants [2]. In plants, the number of genes encoding peptides is higher than that in animals [28]. According to the record on the PlantPepDB database (http://www.nipgr.ac.in/PlantPepDB/), to date there have been 3848 plant-derived peptides identified from 443 plant species including two cryptogams (i.e., algae and bryophyte) plus 17 gymnosperm and 424 angiosperm, and this number is still increasing [7]. In model plants, such as rice, *Arabidopsis* and alfalfa, HDPs have been estimated to account at least for 3% of the expressed proteins [1,27,29]. The widespread occurrence and dynamic activity of HDPs underscored their critical role in plant innate immunity [30].

HDPs show a wide diversity in size, sequence, structure and antimicrobial mechanisms. The plant HDPs are apt to evolve dynamically, which often results in the presence of multiple HDPs gene families [30]. Based on the sequence and structure similarity, as well as the number and arrangement of cysteine residues in the primary sequence, plant HDPs can be classified into at least eight major families, including defensins, thionins, non-specific lipid-transfer proteins (LTPs), hevein-like peptides, Snakins, knottins, α-hairpinins, and cyclotides [1,27]. A search for potential AMPs in 1267 plant transcriptomes by a computational pipeline resulted in 4849 sequences assigned to the Snakin family, showing that the Snakin family peptide is the most abundant potential AMP that is active against fungal and bacterial pathogens [31]. Herein, this review is devoted to the novel cysteine-rich plant HDPs families of Snakins, describing the general characters of the Snakin family, as well as the individual features of different family members. Specifically, the sequence properties, spatial structures, expression patterns and biological activities are described. This provides better understanding of the versatile biochemistry and molecular properties of the Snakin/GASA family peptides for biotechnological application.

## 2. Molecular Characterization of Plant Snakins

Snakins are generally small (~7 kDa), positively charged and cysteine-rich proteins [32] involved in plant defense responses, such as antimicrobial activity against a wide range of phytopathogens [33,34,35,36,37,38,39,40] and animal pathogens [41,42], as well as in a variety of plant development processes [43,44,45,46,47]. The first defined Snakin peptide, Snakin-1 (StSN1) was purified from potato tubers by Segura et al., (1999), who reported that it had some sequence motifs in common with snake venoms and named it Snakin [40]. Thereafter, accumulated studies have been implemented for Snakins, and now it is known that the Snakin family peptides are characterized by having 12 cysteine residues at constant positions in a conserved domain called GASA (Gibberellic Acid Stimulated in *Arabidopsis*) at the C-terminal region. They also have a putative signal peptide at the N-terminus, and a variable region in the middle of their sequences. The amino acid sequence of GASA domain in Snakins consist of a peculiar Cys-motif “XnCX_3_CX_2_RCX_8(9)_CX_3_CX_2_CCX_2_CXCVPXGX_2_GNX_3_CPCYX_10(14)_KCP” (where X is any of 20 proteinogenic amino acid residue except for cysteine, R is arginine, V is valine, P is proline, G is glycine, Y is tyrosine and K is lysine), in which the number and arrangement of cysteine residues is highly conserved [7,27,29,32,48]. As these features are also shared by GASA gene family members, thereafter we use Snakin/GASA instead of Snakin in this review. Comparison with the other major families of the aforementioned plant HDPs which have less than ten cysteine residues [27], the Snakin/GASA family peptides represent the foremost cysteine-rich peptides among different class of plant antimicrobial peptides. HPLC-ESI-QTOF and crystallography analyses show that the 12 highly conserved cysteine residues are involved in the formation of up to six disulfide bonds [49,50]. The 3D structure by X-ray and mass spectrometry data unravels a helix-turn-helix (HTH) motif conserved in the Snakin peptides [50,51]. These results suggest that the disulfide bonds and the HTH motif are necessary for the spatial structure of Snakin/GASA and might be critical for the Snakin’s interactions with its target (e.g., cell membrane, protein and DNA).

The Snakin/GASA peptides comprise a multigene family and are distributed in a vast number of plants, yet they are not present in animals. Although the homologous gene sequences can also be found in a few bacteria, including *Escherichia coli*, *Klebsiella pneumoniae*, *Nitriliruptoraceae bacterium*, *Acinetobacter baumannii*, *Soehngenia saccharolytica*, *Glycocaulis profundi* and *Staphylococcus warneri* (https://www.ncbi.nlm.nih.gov/), whether or not these genes code for Snakin/GASA peptides requires future investigation. In an early study, by comprehensive genome sequence analysis, approximately 445 genes coding for Snakin/GASA proteins have been discovered in 33 plant species [29]. Further bioinformatics mining data reveals that the Snakin/GASA genes are present in all well-characterized sequenced plant species, but are completely absent in moss and green algae, implying that the emergence of Snakin/GASA could be an adaptation of ancestral plants to land [52]. An overview of the Snakin/GASA family members in some selected plant species (Table 1) reveals that the Snakin/GASA peptides exhibit significant diversity in many aspects, such as the number of family members, protein length and pI values (Table 1).

## 3. Synthesis and Distribution of the Snakin/GASA

The potato StSN1 (Snakin-1) and StSN2 (Snakin-2) peptides are so far the most extensively studied members of the Snakin/GASA family. Both StSN1 and StSN2 are purified peptides obtained from a crude cell wall extract from potato tubers, but they show only 38% sequence similarity [35,40]. Like most peptides identified up to now, the Snakin/GASA family peptides are derived from a longer nonfunctional precursor [60]. The mature peptide of St-SN1 (Uniprot: Q948Z4) carrying 63 amino acid residues, is derived from a preprotein (Genbank accession: AJ320185) of 88 amino acids [40], whereas StSN2 (Uniprot: Q93X17) carrying 66 amino acid residues, is from a preproprotein (Genbank accession: AJ312424) of 104 amino acids [35], harboring a 15-amino acid region between the N-terminal signal sequence and the mature StSN2 sequence [38,55]. Despite the recognition of their precursor proteins, whether or not StSN1 and StSN2 can be processed in vivo remains to be determined. Anyhow, imaging and immunological analyses showed that the N-terminal signal peptide of *Arabidopsis* AtGASA4 and 6 was cleaved in planta [61], confirming that the in vivo cleavage can occur. Further sequence analyses of different genes reflect that almost all of the Snakin/GASA family members have a N-terminal signal peptide, suggesting that they are a class of secreted proteins [32,55,62]. 

The Snakin/GASA peptides, since their first isolation and functional characterization in potato, have raised substantive research concerns. As a consequence, a genome-wide analysis of the Snakin/GASA genes has been performed. The exist of a large multigene family (18 members) with divergent expression patterns and antimicrobial spectrum in the potato species have been uncovered [55]. Likewise, a growing number of gene sequences of the Snakin/GASA family members have been identified in many well-sequenced plant species, including 37 members in both common wheat [57] and soybean [58], twenty-six members in the apple tree (*Malus domestica*) [53], sixteen members in the rubber tree (*Hevea brasiliensis*) [56], fifteen members in *Arabidopsis* [53], fourteen members in the grapevine (*Vitis vinifera* L.) [59], ten members in maize and nine members in rice [47] (Table 1). It has to be noted that the peptide length of the above identified Snakin/GASA members varied remarkably (ranging from 64 to 1099 aa) (Table 1), and the gene sequence of the GASR members in the common wheat is particularly longer (>261 aa) than that in the other plant species (generally consist of 80–120 aa) [62]. Besides, the number of family members (between 5 and 37) and pI values (from 4.11 to 10.14) also vary widely among different plant species and individual members, respectively. This considerable discrepancy addresses the necessity for further structural and functional characterization of the Snakin/GASA genes to elucidate their biological relevance, despite of their high sequence similarity. As HDPs are often referred to as proteins smaller than 100 amino acids [2], therefore in this review special focus is put on the typical Snakin/GASA peptides whose length (precursor or the mature form) is below this upper limit. The biochemical properties, gene expression patterns, and biological activities of some representative members are described (Table 2).

As the subcellular localization provides key information to identify the protein function, a line of experiments has been attempted to determine the in planta location of the Snakin/GASA genes. Transient expression of the rice OsGASR-GFP fusion proteins in onion epidermal cells show that both OsGASR1 and 2 are primarily distributed to the cell wall or apoplast [45,73]. The *Arabidopsis* GASA5 is proved to be located in the cell wall or extracellular matrix in both transiently and stably transformed plants [69]. The cell wall-localization of Snakin/GASA genes has also been illustrated by immunoblot analysis of the petunia GIP2 proteins [43] and the gerbera GASA members [74] in different cell fractions from *Petunia hybrida* and *Gerbera hybrida*, respectively. In addition to these observations, some Snakin/GASA proteins (e.g., the soybean GsGASA1 [75]) have been found not only in the cell wall but also in the cytoplasm and nuclei; whereas some other Snakin/GASA proteins are not located to the cell wall at all, even though they bear an N-terminal signal peptide. For instance, the rubber tree HbGASA5 and HbGASA9 proteins are distributed in the nucleus and throughout the cytoplasm [56]. The fluorescent signal of the citrus CcGASA4::GFP fusion protein is observed in the nucleus and plasma membrane [76]. The potato StSN1::GFP fusion proteins are found to be throughout the plasma membrane of the agroinfiltrated leaves in *Nicotiana benthamiana* [64]. Noteworthy, the subcellular localization assay of StSN1 in transient expression insect cells shows that the peptide is heterogeneously restricted in the cytoplasm. Nevertheless its mature form (lacking the signal peptide) is conspicuously present in the nucleus of the infected insect cells, even though StSN1 has no potential nuclear localization signal (NLSs) [34]. The nucleus-localized mature StSN1 has also been observed in transient transgenic tobacco cells, although the fluorescence signal is very weak [64]. Likewise, the *Arabidopsis* AtGASA4 and AtGASA6 are generally present at the cell periphery, but they have been visualized to localize in the nucleus when the signal peptides are lacking [67]. In addition to the above subcellular distribution, AtGASA4 contains a non-cleavable signal peptide and is speculated to attach to the endoplasmic reticulum (ER) [77]. Moreover, the petunia GIP1 is confirmed to locate within the ER when expressed in tobacco BY2 cells [43]. However, the biological function of the putative ER-retained proteins still needs further exploration. In conclusion, the subcellular localization of Snakin/GASA varies (i.e., cell wall, plasma membrane, nucleus, cytoplasm and endoplasmic reticulum) amongst the different family members, and their transition between cell periphery and nucleus might be of great importance to their antimicrobial function.

## 4. Spatiotemporal Expression of the Snakin/GASA

The Snakin/GASA family members show divergent expression patterns in regard to spatial and temporal regulation. In potato, the transcripts of *StSN1* are found to be particularly abundant in axillary, stem, floral buds, and in fully developed petals, nevertheless no expression has been detected in roots, stolons or leaves [40,65]. Moreover, the *StSN1* promoter expressed mainly in young tissues and zones (e.g., shoot apex, apical bud, vascular stem and root tissues, etc.) associated with vigorous growth and cell division, and it is active in young stages gradually decreasing as the plant ages. Accordingly, at the protein level StSN1 is present mainly in young tissues associated with active growth and cell division zones [65]. The steady-state mRNA levels of *StSN2* are high in tubers, flowers, roots and leaves [35,55]. The potato *Snakin-3* is expressed in roots, stolons, stems and axillary buds [55]. In rice, the Snakin/GASA homologs gene, *OsGASR* are highly expressed in panicles, shoot apical meristem (SAM), moderately in roots and young leaves, but not present in mature and flag leaves [45]. The expression of *OsGASR1* and *2* are relevant to cell proliferation in meristems and panicles development [45]. In alfalfa, the *MsSN1* expression was detected in all tissues analyzed, including roots, stems, leaves and young floral buds [52]. In the *Pará* rubber tree (*Hevea brasiliensis*), the *Snakin-1* is predominantly expressed at the early stages of leaf development [78]. In *Arabidopsis*, the *AtGASA14* gene is expressed in young leaves and the elongation zone of roots [46], the *AtGASA4* promoter directed GUS is stained predominantly in vegetative shoot apical meristems and imitating leaves [54], in contrast, staining for *AtGASA5* promoter is detected in the root hairs, the basal portion of the roots, the shoot apex, and the inflorescent meristems [69]. In *Peltophorum dubium*, the first isolated Snakin-like gene *PdSN1* is strongly expressed during seedling development, which is 40 fold higher than adult leaves [51]. In cucumber the *Snakin* gene homologous to *Arabidopsis AtGASA11* (At2g18420) is expressed in the late stage of fruit development [79]. In maize, the in situ hybridization experiment shows expression of *ZmGSL2*, *4*, *6* and *9* in emerging lateral root primordia, confirming a role of Snakin/GASA genes in lateral root development [47]. In grapevine, transcript levels of *VvGASA1* and *2* are found to be high in leaves, whereas *VvGASA9* and *10* are abundant in fruits and seeds [59]. Altogether, these results show that the Snakin/GASA genes are expressed in both tissue- and developmental-specific manner, and most of the them are highly expressed in young plant tissues/organs and vigorous growth site, or in reproductive and storage organs, signifying their role in plant growth and development, as well as being a first line of defense barrier.

## 5. Hormonal Regulation of the Snakin/GASA Genes

The *Snakin/GASA* family genes have been reported to be modulated by gibberellin (GA), abscisic acid (ABA) and other phytohormones (see reviews [32,62,80]). Most of them are responsive to exogenous GA treatment, for example, transcripts of *Arabidopsis AtGASA4*, *6*, *7*, *8* and *13* [66,67], rice *OsGASR1*, *2* and *OsGSR1* [45,73], petunia *GIP1*, *2*, *3* and *4* [43], maize *ZmGSL1*, *2*, *4*, *6* and *9* [47], *Salvia miltiorrhiza SmGASA4* [72] are upregulated by GA. On the contrary, other Snakin/GASA members like *Arabidopsis AtGASA1*, *5*, *9* and *11* [67], potato *StSN2* [35] are repressed by GA. However, some genes such as *Arabidopsis AtGASA2*, *3*, *10*, *12*, *14* and *15*, potato *StSN1, StSN2* and *Snakin-3* [40,55], their expression levels are not altered by GA [67]. Interestingly, exogenous application of GA induced *GsGASA1* expression in leaves but inhibited it in roots of the soybean [75]. Similarly, GA treatment induces the *AtGAST1* expression in meristem tissues but represses it in roots and leaves in *Arabidopsis* [77]. These findings demonstrate that the *Snakin/GASA* genes have tissue-specific responses towards GA application.

ABA is also an important hormone that interacts with the *Snakin/GASA* family genes. ABA can induce the expression of *AtGASA2*, *3*, *5* and *14*, but inhibits the expression of *AtGASA7* and *9* in *Arabidopsis* [67]. In potato, the expression of *StSN2* is induced by ABA, but *Snakin-3* is downregulated by ABA treatment; nevertheless the expression level of *StSN1* is not regulated by ABA [35,55]. Additionally, several Snakin/GASA family members, such as *GAST1* (Shi et al., 1992), *Snakin-2* [35], the beechnut (*Fagus sylvatica*) *FsGASA4* [81], and *GsGASA1* [75] are regulated antagonistically by GA and ABA. This antagonistic activity may provide a mechanism for fine-tuning developmental processes, such as growth, flowering and germination. 

Aside from responsiveness to GA and ABA, it has been found that *OsGSR1* directly regulates the brassinosteroid (BR) biosynthesis and signaling pathway [59,73]. The *Snakin-1* silenced transgenic potato plants causes accumulation of reactive oxygen species (ROS), significant reduction of ABA, increase of salicylic acid (SA) and GA and downregulation of sterol biosynthesis [65](. The transcript of *HbGASA7-1*, *14* and *16* is significantly upregulated after the treatment with ethylene (ETH), SA, or jasmonic acid (JA) [56]. These results suggest that the *Snakin/GASA* genes play essential roles in redox balance and participate in a complex crosstalk among different hormones. 

## 6. The Role of Snakin/GASA Involved in Plant Growth and Development

Accumulated evidence confirmed that the *Snakin/GASA* family members are involved in a variety of plant physiological processes, such as cell division, floral induction, seed germination and root growth. Silencing of potato *St-GSL1*(*StSN1*) resulted in plants with smaller leaves and affected cell division, metabolism, and cell wall composition of leaves [64]. Suppression of both *AtGASA4* and *6* results in late flowering. Accordingly, overexpression of *AtGASA6* leads to early flowering in *Arabidopsis* [61], whereas transgenic overexpression in parallel with suppression of *AtGASA4* clearly show its involvement in bolting, branching, flowering and seed development in *Arabidopsis* [54]. Moreover, *Arabidopsis* AtGASA4 and AtGASA14 proteins can interact with the cell membrane-localized receptor-like kinase protein VH1/BRL2 participating in the veins’ development [82]. While *Arabidopsis AtGASA4*, *6* and *14* motivate plant development [46,61,66], *AtGASA5* is known as a negative regulator of GA-induced flowering and stem growth [54]. Besides, overexpression of *FaGAST1* in strawberry causes growth delay [44]. 

Apart from the above physiological function, the Snakin/GASA family members also become involved in some abiotic stresses. In *Arabidopsis*, overexpression of *AtGASA14* can promote salt stress resistance [46], and *AtGASA5* is responsive to heat stress by regulating the SA signaling and the accumulation of heat shock protein [67]. Heterologous expression of the beechnut *FsGASA4* in *Arabidopsis* improves plant resistance to salt, oxidative and heat stress [81]. In the same way, heterologous expression of *S. miltiorrhiza SmGASA4* in *Arabidopsis* promotes flower, root development and enhances plant resistance to salt, drought, and paclobutrazol (PBZ) stress. The *SmGASA4* also displays a role in the biosynthesis of secondary metabolism [72]. Additionally, the Snakin/GASA family members can serve as antioxidants and influences ROS accumulation [56,66,68]. Taken together, Snakin/GASA may act as a polypeptide signal or the second messenger affecting plant growth and development [62].

## 7. The Role of Snakin/GASA Involved in Plant Innate Immunity

Snakin/GASA is one of the most important types of plant HDPs, as they can inhibit a wide range of bacterial and fungal growth at extremely low concentrations. The typical examples are the Snakin/GASA family members originate from the potato plants, namely StSN1, StSN2 and Snakin-3. Specifically, the purified StSN1 peptide is shown in an in vitro challenge experiment to be toxic to fungal pathogens like *Fusarium solani, Fusarium culmorum*, *Bipolaris maydis* and *Botrytis cinerea*, and many bacterial pathogens such as *Clavibacter michiganensis* subsp. *Sepedonicus*, at extremely low concentrations (EC50 <10 μM) [34,36,37,40,83]. Moreover, overexpression of *StSN1* in potato [33,38] and wheat [39] improves plant resistance to commercially important pathogens, including *R. solani*, *E. carotovora* and *Gaeumannomyces* *graminis*, confirming its in vivo antimicrobial activity. Consistent with StSN1, both the alfalfa MsSN1 and *Solanum chacoense* Snakin-1 have been demonstrated in vitro and in vivo to have antimicrobial activity against many fungal (e.g., *Phoma medicaginis*, *Colletotrichum trifolii* and *Blumeria graminis* f.sp. *tritici*.) and bacterial pathogens (e.g., *Agrobacterium tumefaciens*, *Sinorhizobium meliloti* and *Pseudomonas fluorescens*) [52,84]. Meantime, StSN2 is shown to be active against many Gram-negative bacteria, Gram-positive bacteria and fungal species (EC50 = 0.1~30 μM) [35,49]. Overexpression of the tomato *snakin-2* (*SN2*) genes enhances the tolerance of transgenic plants against *C. michiganensis* [70], while silencing the *snakin-2* gene in tobacco plants increased the susceptibility of plants to *C. michiganensis* [85]. Additionally, a recent study revealed that the potato Snakin-3 is probably associated with plant defense, as its gene expression levels are remarkably increased upon pathogen infection [55]. Intriguingly, the purified peptide Snakin-Z from *Ziziphus jujuba* fruits displays significant antimicrobial activity against fungi, such as *Phomopsis azadirachtae* with the minimal inhibitory concentration (MIC) value of 7.65 mg/mL. Nevertheless, it shows no negative effects on human red blood cells. The feature of high potent antimicrobial activity but low hemolytic activity of Snakin-Z leads to a potential therapeutic application of the Snakin/GASA peptides in human [71].

Besides its antifungal and antibacterial activity, the Snakin/GASA family genes also have an assignable role in protecting plants from virus and nematodes. Over-expression of *GmSN1* enhances virus resistance in both *Arabidopsis* and soybean [86]. The citrus homolog gene of *AtGASA4*, *CcGASA4*, is highly induced in citrus leaves after infection with *Citrus tristeza virus* [76]. Furthermore, the pepper CaSn protein has been reported to participate in defense of plants against nematodes (*Meloidogyne* spp.) [87].

## 8. Snakin/GASA in Biotechnology

The intriguing biological activities of Snakin/GASA make them attractive biotechnological targets, especially for the development of novel disease control agents [32]. Several heterologous expression approaches have been attempted to produce Snakin/GASA peptides with antifungal and/or antibacterial applications. Overexpression of *StSN1* and *StSN2* under a potato light-inducible *Lhca3* promoter in potatoes, leads to resistance to blackleg disease without changes in plant morphology [38](. In agreement with this result, overexpression of the Snakin/GASA of different plant origin in different plant species has been evidenced to confer broad-spectrum resistance to a wide variety of invading phytopathogens and virus [33,38,39,52,70,85,86].

In addition to the transgenic expression approach, recombinant Snakin/GASA produced in *E.coli* [36,37,39,49,51,88], yeast (*Pichia pastoris*) [37], insects [34], and chemically synthesized Snakin/GASA [83] also show promising application in agronomy. Recently, a synthetic Snakin-1 designed from the natural form of potato StSN1 has been reported to exhibit significant inhibitory effect against a number of food spoilage yeast, but has no adverse safety concern on human consumption [83], suggesting great potential applicability of the Snakin/GASA peptides in protecting food, pharmaceuticals, or cosmetics from decomposition by microorganisms. 

## 9. Proposed Mechanisms of Action of Snakin/GASA

Even though many of the Snakin/GASA genes have been characterized and confirmed to have different biological functions, their mechanism of action has not been completely elucidated. In this review, we summarize a couple of hypotheses to explain the function mechanism of the Snakin/GASA. One hypothesis is that the high cationic nature of the Snakin/GASA allows it to interact with the negatively charged component of its site of action, which then results in destabilizing (or interacting with) the target and promoting immune response [9,30,83,89]. This hypothesis is supported by the finding that the crystal structure of the Snakin/GASA peptide displays a large positive electrostatic surface with a pronounced electrophile cleft [50,51]. However, so far the site of action of the Snakin/GASA family peptides remains elusive. Previously, it has been proposed that the negatively charged membrane component of pathogens is the target of the Snakin/GASA [3,50]. Consistent with this conception, it is shown that the antimicrobial peptides StSN2 exhibits a non-specific pore-forming effect in the membrane of bacteria, fungi and *N. tabacum* protoplasts [49,89]. In addition, some recent studies indicate that the Snakin/GASA family peptides may also target DNA, the most negatively charged polymer in nature [90], exerting its antimicrobial activity probably by deregulating the microbial gene expression [51]. Support to this notion includes the finding that the Snakin/GASA peptides contain a HTH motif which is a well-established motif found in many proteins, such as transcription factors, known to bind to the major groove of DNA [51]. Furthermore, the nuclear-localization of natural StSN1 peptide (lacking the signal peptide) also agrees with this assumption [34]. 

Another hypothesis is that the Snakin/GASA exerts its function through a signaling transduction pathway (refer to the review [62]). The Sankin/GASA peptides have been considered as phytohormonal signaling transducer and integrator, tightly linked to the biosynthesis and transduction processes of phytohormones, such as the aforementioned GA, ABA and BR. Given the fact that they have putative redox- active sites (cysteine residues), and directly control over the ROS status in plant [46,66,68,91], the Snakin/GASA proteins are likely executing their physiological function through the redox and hormonal signaling pathway [65]. 

Additionally, some unexplored protein-protein interactions might also be involved in the mode of action. Genetic, physiological and bioinformatics analysis of *Pseudomonas* mutants resistant to MsSN1 revealed that bacterial adhesion protein lapA is involved in MsSN1 cell surface attachment or in cell–cell interactions [92]. It has also been reported that the Snakin/GASA peptides mediate bacterial cell aggregation/agglomerating [35,40,49], which may in vivo impede pathogen migration to uninfected areas [40]. Nevertheless, all these hypotheses about the mode of action clearly deserve future studies for confirmation, which are beyond the present contribution.

## 10. Summary and Future Scope

The Snakin/GASA peptides are widely distributed in land plants. Evidence from a large body of in vivo and in vitro expression and activity analysis confirms that the Snakin/GASA family members have potent antifungal and antibacterial effects; meanwhile, they also display dynamic roles in many aspects of plant growth and development process. These outstanding properties give them great biotechnological potential in the fields of pathology and agronomy. Despite the progress in understanding of the important role of Snakin/GASA family genes to plants, there are still a few questions to be addressed.

Firstly, a clear classification of the Snakin/GASA family peptides: Up to now, the most distinguishable molecular feature of the Snakin/GASA peptides is the presence of 12 cysteine residues at the conserved position within an approximately 60 amino acid domain in their protein sequence. According to this criterion, at least 445 genes have already been discovered as Snakin/GASA peptides [29]. Nevertheless, except for the StSN1 [40] and StSN2 [35] from potato and the Snakin-Z from *Z. jujube* [71], the rest of the Snakins were discovered through genome analyses rather than through protein isolation and characterization. Additionally, except for this given similarity in gene sequences, divergent variations in amino acid composition, sequence length, expression pattern and functionality exist, preventing more detailed classification of these genes. Since peptides are often referred to as proteins smaller than 100 amino acids [2], the identified family members whose length is over this range might be considered as the Snakin outgroup. However, such a criterion is rather brutal; a more precise standard should be utilized to discriminate the Snakin type of HDPs from GASA proteins. Previously, the specific cysteine pairs are considered to be critical for Snakin’s structure and activity [32,48]. However, recent findings indicate that the disulfide bonds may not be essential for their antimicrobial function [41,51]. This further complicates the attempts to distinguish the Snakin from other peptides with the same pattern of 12 conserved cysteine residues, like the genes from bacteria *E. coli*, *K. pneumoniae*, and *N. bacterium*. as described before. Future in-depth structural and functional confirmations are thus required to explore some more significant and specific characteristics of the Snakin peptides.

Secondly, clarification of the functional mechanism: although different hypotheses have been proposed to explain the mode of action of the Snakin family peptides, our knowledge on the biochemical and molecular reaction dynamics of Snakin is still limited. A more accurate action site, target and working model of the Snakin need to be determined.

Thirdly, functional diversity and redundancy: The occurrence of a considerable number of the Snakin/GASA family members raise the question of whether they all have similar biological functions or just share similar sequence information, but are involved in diverse biological processes. The various spatiotemporal expression patterns of different Snakin homologs are more inclined to the function diversification. However, currently most functions of Snakins are deduced from gene expression at the gene level, rarely from direct proof of peptide activity. What is more, except for a few plant species such as potato, the major function of the Snakin/GASA in most of the plant species revealed so far is focus on plant growth and development. Few efforts have been invested in confirming their immune function in plant defense response, which clearly deserves special attention afterward.

Last but not least, despite the increasing reports of potential applications, the number of approved HDPs is low. Application of Snakins is facing various challenges, for example, safety considerations including immunogenicity or cross-reaction with other host receptors such as neuropeptide and peptide hormone receptors [12,14]. Although resistant mutants to HDPs are likely to develop slowly compare to antifungal molecules interacting with a single site, the mutant of yeast show resistance to the defensin family HDPs has been documented [93]. Another aspect which has to be taken into account for the prospective application of Snakins is allergy, as a recent report shows that GRPs are clinically relevant plant allergens [94]. Altogether, the mode of action of Snakins should be demonstrated and caution should be taken before agriculturally and clinically applying Snakins.

## Figures and Tables

**Table 1:** Overview of the Snakin/GASA gene family in some selected plant species.

Plant Species	Family Members	Length (aa)	pI	References
Petunia (*Petunia hybrida*)	5	104–112	9.08–9.40	[43]
*Arabidopsis thaliana*	15	87–275	7.41–9.98	[47,53,54]
Rice (*Orazy sativa*)	9	92–152	8.77–9.28	[47]
Maize (*Zea mays*)	10	80–129	8.26–9.30	[47]
Potato (*Solanum tuberosum*)	18	88–143	6.01–9.72	[55]
Apple (*Malus domestica*)	26	88–305	4.11–10.14	[53]
The rubber tree (*Hevea brasiliensis*)	16	88–241	8.75–10.00	[56]
Common wheat (*Triticum aestivum* L.)	37	261–1099	4.99–5.27	[57]
Soybean (*Glycine max*)	37	66–198	5.65–9.54	[58]
Grapevine (*Vitis vinifera* L.)	14	64–298	8.50–9.64	[59]

**Table 2:** Diversity of Snakin/GASA peptides of different plants.

Name	Uniprot/Gene Locus	Length (aa)	N-Terminal Signal Peptide (aa)	pI	Origin Species (Common Name)	Subcellular Localization	Expression	Biological Activity	References
Snakin-1/StSN1/GSL1	Q948Z4/EF206292	88	25	8.97	*Solanum tuberosum* (potato)	Plasma membrane, nucleus	Particularly high in axillary buds and stems	Antibacterial and antifungal; Affect ROS and ABA levels; Influence cell division, leaf metabolism and cell wall composition	[33,36,39,40,55,63,64,65]
Snakin-2/StSN2/GSL2	Q93X17	104	23	9.16	*Solanum tuberosum* (potato)	Cell wall	High in tubers, flowers and leaves	Antibacterial and Antifungal	[35,38,55]
Snakin-3/Protein RSI-1	XP_006364638	96	29	9.32	*Solanum tuberosum* (potato)	nd	Root, stolon, stem	Antibacterial and Antifungal	[55]
AtGASA4	AT5G15230	106	25	9.46	*Arabidopsis thaliana*	Cell periphery, Nucleus	In all meristematic regions-in shoots, in flower buds, in primary and lateral roots	Promote lateral root formation; Positively regulate seed size and products; Reduce ROS accumulation	[54,66,67]
AtGASA5	AT3G02885	97	27	9.68	*Arabidopsis thaliana*	Cell wall, or extracellular matrix	Root without apex	Suppress GA-induced germination; A regulator of flowering time and stem growth; May be metalloproteins having redox activity	[68,69]
AtGASA6	AT1G74670	101	23	9.01	*Arabidopsis thaliana*	Cell periphery, nucleus	Flower, silique, early stage of germinated seeds	Overexpression leads to early flowering; Upregulated by GA	[61]
Tomato Snakin-2	ADR32106	104	38	9.06	*Solanum lycopersicum* (tomato)	nd	nd	Antibacterial	[70]
MsSN-1	AFE82743	91	25	9.20	*Medicago sativa* (Alfalfa)	nd	Higher in leaves than in roots	Antibacterial and Antifungal	[52]
Snakin-Z	nd	31 (incomplete sequence)	nd	8.76	*Ziziphus jujube* (Jujuba)	nd	nd	Antibacterial, Antifungal and Antioxidant; having no haemolytic activity	[71]
SmGASA4	CL13560Contig1	110	nd	nd	*Salvia miltiorrhiza*	nd	nd	Promote flower and root development; Enhance plant resistance to salt, drought; Secondary metabolism biosynthesis	[72]
OsGASR1	BAD67542	93	27	8.77	*Oryza sativa* (rice)	Apoplast or cell wall	Strongly expressed in florets, root apical meristem (RAM) and shoot apical meristem (SAM),	Involved in panicle differentiation	[45]
OsGASR2	BAD67543	105	23	8.78	*Oryza sativa* (rice)	Apoplast or cell wall	Expressed in both florets and branches, root apical meristem (RAM) and shoot apical meristem (SAM),	Involved in panicle differentiation	[45]
OsGSR1	AAT42201	110	28	9.28	*Oryza sativa* (rice)	Plasma membrane, cytoplasm and nucleus	Expressed prominently in young and actively growing organs	Influence brassinosteroid signaling	[73]

nd, not determined yet.
